# Dexmedetomidine ameliorates cognitive and affective deficits by modulating neuroinflammation and neurogenesis in an Alzheimer’s disease mouse model

**DOI:** 10.3389/fnagi.2025.1724739

**Published:** 2025-12-15

**Authors:** Mai Li, Chanyuan An, Xin Wang, Minghe Ren, Shiyu Liu, Ruixin Chen, Yuyan Guo, Jun Wang, Yulang Fei, Dafei Ma, Kaige Ma, Yuming Zhang

**Affiliations:** 1 Department of Anesthesiology, Shaanxi Provincial People’s Hospital, Xi'an, China; 2 Institute of Neurobiology, School of Basic Medical Sciences, Xi’an Jiaotong University Health Science Center, Xi’an, China; 3 Department of Radiation Oncology, Second Affiliated Hospital of Xi’an Jiaotong University, Xi’an, China; 4 Department of Anesthesiology, Shaanxi Provincial Cancer Hospital, Xi’an, China; 5 Department of Neurology, The First Affiliated Hospital of Nanyang Medical College, Nanyang, China; 6 Zhang Zhongjing Chinese Medical Research Institute of Nanyang Medical College, Nanyang, China; 7 Golden Horse Arts & Crafts Co., Ltd, Zhejiang, China

**Keywords:** Alzheimer’s disease, dexmedetomidine, neuroinflammation, neurogenesis, cognition

## Abstract

Alzheimer’s disease (AD) involves progressive cognitive decline and neuropsychiatric symptoms that are strongly linked to neuroinflammation and aberrant hippocampal neurogenesis. We examined whether dexmedetomidine (Dex), a clinically used selective α_2_-adrenergic agonist, could mitigate Aβ_1–42_–induced pathology in mice. After intracerebroventricular Aβ_1–42_ injection, animals were treated with Dex (25 or 50 μg/kg/day) for 7 days; a subgroup additionally received the α_2_ antagonist Yohimbine. Behavioral tests showed improved memory performance across recognition and spatial paradigms, accompanied by reduced anxiety-like behavior in exploratory assays. Histological analyses with Nissl and doublecortin (DCX) staining indicated preserved neuronal integrity, fewer degenerating cells, and normalization of pathological neurogenesis. At the molecular level, Dex suppressed the expression of pro-inflammatory and apoptotic genes (CXCL2, IL-1β, iNOS, SPHK1) and lowered hippocampal malondialdehyde, consistent with reduced oxidative stress and improved cellular resilience. Yohimbine partly reversed these effects, supporting α_2_-adrenergic involvement but leaving open the possibility of additional pathways contributing to the response. Overall, our results suggest that Dex protects against Aβ-driven injury through coordinated regulation of neuroinflammation, oxidative stress, and neurogenesis, underscoring its promise as a molecularly targeted candidate for early therapeutic strategies in AD management.

## 1 Introduction

Alzheimer’s disease (AD) is a progressive, irreversible neurodegenerative disorder characterized by memory decline, cognitive impairment, and emotional disturbances, representing the leading cause of dementia in the aging population. Despite significant research efforts targeting the core pathological features such as neuron loss, amyloid-*β* (Aβ) plaque accumulation, and neurofibrillary tangles, no curative treatments have been developed.

Alzheimer’s disease remains a critical public health issue globally. According to the China Alzheimer’s Disease Report 2024, an estimated 16.99 million people in China were affected by AD or other dementias in 2021, with a prevalence rate of approximately 2.8%. These figures highlight a substantial societal burden and the need for novel neuroprotective strategies.

Dexmedetomidine (Dex), a selective α_2_-adrenergic receptor agonist, is widely used in clinical practice for its analgesic, anxiolytic, and sympatholytic effects (Zhai et al., 2023). These receptors are located at both presynaptic and postsynaptic sites within the central nervous system. Dex has been shown to partially alleviate postoperative cognitive dysfunction (An et al., 2020). Previous research demonstrated that Dex alleviates both cognitive and affective impairments in AD mouse models, possibly through the modulating neuroinflammatory pathways (Ma et al., 2024).

Emerging evidence suggests reduced hippocampal dendritic spine density and impaired neurogenesis in both human and murine AD models (Fernandes et al., 2015; Kreutzmann et al., 2015). Although Dex has been associated with cognitive improvements (Su et al., 2016), its impact on adult neurogenesis in AD remains under explored. Adult hippocampal neurogenesis is crucial for learning, memory, and cognitive flexibility, occurring throughout life in the dentate gyrus, where the neural stem cell (NSCs) retains self-renewal capacity (Gonçalves et al., 2016; Seong et al., 2016; Niklison-Chirou et al., 2020). This process facilitates synaptic remodeling, spatial memory, and behavioral adaptation (Lieberwirth et al., 2016).

Inflammatory signaling alters the proliferation and differentiation of NSCs in AD (Sung et al., 2020). Therapeutic approaches that restore hippocampal neurogenesis *via* anti-inflammatory and antioxidant mechanisms have demonstrated cognitive benefits in adult animal models (Tan et al., 2022; Zeng et al., 2023). Building on these findings, Dex may promote neurogenesis in the subgranular zone (SGZ) of the AD hippocampus by modulating neuroinflammation (Vadodaria et al., 2017; Hu et al., 2022; Wang et al., 2022). Therefore, we hypothesized that Dex may attenuate cognitive and emotional deficits in AD by modulating hippocampal neuroinflammation and aberrant neurogenesis. To test this, we established an AD mouse model via bilateral intracerebroventricular injection of oligomeric Aβ_1–42_ and administered Dex intraperitoneally. It is important to note that the acute Aβ₁–₄₂ intracerebroventricular injection model captures early amyloid-driven neurotoxicity but does not recapitulate the progressive tau pathology or chronic neuroinflammation characteristic of the full AD spectrum (Yokoyama et al., 2022). Behavioral tests were performed to assess cognitive and anxiety-like phenotypes, while histological and molecular analyses were conducted to evaluate hippocampal neurogenesis, neuronal integrity, and inflammatory markers.

## 2 Materials and methods

### 2.1 Animal model

Experiments were conducted using two-month-old male C57BL/6 J mice weighing 25–30 g. The animals were housed in groups of five per cage under controlled conditions, with a temperature of 22 ± 2 °C, a 12-h light/dark cycle with lights on from 08:00 to 20:00, and 50–60% relative humidity. Throughout the experiment, animals had continuous access to food and water. The Institutional Animal Care and Use Committee of Xi’an Jiaotong University Health Science Center reviewed and approved all experimental protocols, ensuring full compliance with guidelines to minimize animal distress (Approval No.: XJTUAE2024-28, Date: 2024-02-26). Mice were obtained from Xi’an Jiaotong University’s Laboratory Animal Center. Sample sizes were informed by prior studies using similar Aβ₁–₄₂–induced AD models, in which comparable behavioral, histological, and molecular outcomes have yielded robust and reproducible effect sizes. Experiments were performed in at least three independent batches, and animals were randomly assigned to groups. Final sample sizes were selected to ensure adequate statistical power while adhering to the “3Rs” principles. All behavioral assessments, tissue processing, imaging analyses, and statistical evaluations were conducted by investigators blinded to group allocation.

Animals were randomly allocated into four experimental groups using a random number generator to minimize selection bias: Control, AD, AD + Dex at 25 μg/kg, and AD + Dex at 50 μg/kg. Anesthesia was induced via intraperitoneal administration of 1.25% (w/v) tribromoethanol (Avertin, T48402, Sigma-Aldrich) in saline at 250 mg/kg. Once fully anesthetized, mice were secured in a stereotaxic apparatus for subsequent procedures. Stereotaxic injection procedure was followed as previously described (Ma et al., 2018, 2024). Control mice received a 3 μL intracerebroventricular (i.c.v.) injection of sterile saline (coordinates: ML: 1.13 mm, AP: 1.0 mm, DV: 3.0 mm). AD model mice received a 3 μL injection of 100 μM oligomeric Aβ_1–42_ solution. For i.c.v. Aβ_1–42_ injections, mice were anesthetized with 2% isoflurane delivered in oxygen. Analgesia was provided with a single subcutaneous injection of meloxicam (1 mg/kg) prior to recovery.

An established intracerebroventricular Aβ_1–42_ injection protocol was used, as previously described in studies reporting consistent cognitive, synaptic, and neuronal alterations following Aβ_1–42_ administration (Morroni et al., 2016; Ma et al., 2018; Mitsushima, 2025). The same stereotaxic coordinates and injection parameters were used in our previous work (Ma et al., 2024), and the present study followed this validated protocol.

Beginning on the seventh day following Aβ administration, Dex was administered intraperitoneally at doses of 25 or 50 μg/kg for seven consecutive days. Animals were excluded from analysis if they failed to recover from Aβ injection, showed severe illness unrelated to treatment, or if tissue sections were damaged during processing. The number of animals excluded at each stage is reported in the figure legends.

In a separate cohort, AD mice treated with Dex were further divided into AD + Dex and AD + Dex + Yohimbine groups. Mice in the AD + Dex group were given 50 μg/kg Dex daily (i.p.) for a 7-day course, whereas the Yohimbine co-treatment group received 1.25 mg/kg Yohimbine (i.p.) in conjunction with Dex throughout this timeframe. Behavioral tests were conducted on all mice following drug administration.

The doses of Dex (25 μg/kg and 50 μg/kg) and Yohimbine (1.25 mg/kg), as well as the 7-day treatment paradigm following Aβ_1–42_ administration, were selected based on previously published peer-reviewed studies (Jantaratnotai et al., 2003; Conrad et al., 2012; Chen et al., 2022; Zhai et al., 2023; Ma et al., 2024; Gulmez Karaca et al., 2025).

### 2.2 Animal behavior tests

All behavioral assays were conducted in a counterbalanced order across experimental groups to avoid systematic sequence effects. The testing schedule for individual animals was further randomized to minimize potential carryover or learning-related bias.

#### 2.2.1 Y-maze test

The apparatus was composed of three identical arms (30 cm long × 5 cm wide × 12 cm high) positioned at 120° from each other. All mice were introduced into the same designated arm and permitted to move freely throughout the maze for a duration of 6 min. An arm entry was counted when all four limbs entered the arm. A valid spontaneous alternation was defined as sequential entry into three different arms (e.g., ABC, BCA), excluding repetitions (e.g., ABA). The percentage of spontaneous alternation was determined using the formula: Alternation (%) = (Number of alternations/[Total number of entries − 2]) × 100. This test measures short-term spatial working memory.

#### 2.2.2 Marble-burying test

In the marble-burying assay, individual mice were introduced into separate cages lined with a 5 cm layer of corn cob bedding, with 20 marbles evenly distributed in a 4 × 5 grid. The quantity of marbles buried at least two-thirds of their volume was documented following a 30-min period of unrestricted exploration. This test evaluates anxiety-like behavior through natural digging responses.

#### 2.2.3 Open-field test

During the open-field test, each mouse was placed at the arena’s center and allowed to explore for 6 min. Movement was recorded and analyzed to determine total distance, time and distance spent in the central area. These parameters reflect locomotor activity and anxiety-related behavior.

#### 2.2.4 Novel object recognition test

A multi-day habituation phase was conducted prior to object exposure. Each mouse was placed individually into the empty testing arena for 10 min per day across three consecutive days, following established NOR protocols (Antunes and Biala, 2012; Leger et al., 2013). In addition, a 1-h acclimation period was provided immediately before testing. This interval refers specifically to the quiet settling time after the experimenter entered the behavioral room and does not replace the multi-day habituation procedure. Two identical objects were positioned diagonally in an open-field arena, about 8–9 cm from the walls. Animals were allowed 6 min of free exploration before being returned to their home cages. The arena was meticulously sanitized using 75% ethanol to eliminate any olfactory residues. Following a 2 h intertrial interval, a novel object replaced one of the original objects at the same location. Mice were then reintroduced and allowed to explore for another 6 min. Exploration was characterized by the animal positioning its nose within 1 cm of the object, with its head facing the object. The recognition index (RI) was computed using the following formula: RI = Tₙₒᵥₑₗ/(Tₙₒᵥₑₗ + T_f_ₐₘᵢₗᵢₐᵣ), where T(ₙₒᵥₑₗ) and T(_f_ₐₘᵢₗᵢₐᵣ) represent the time spent investigating the novel and familiar objects, respectively. This test assesses recognition memory, a function primarily mediated by the hippocampus.

### 2.3 Morphology study

#### 2.3.1 Nissl staining

Fixed brain tissue was cryoprotected in 30% sucrose and sectioned coronally at 14 μm using a cryostat. Sections were hydrated in distilled water for 3 min, stained with Nissl solution (Solarbio, China) for 10 min, dehydrated through graded ethanol, cleared in xylene, and mounted with neutral resin. Neuronal morphology was examined under a light microscope.

#### 2.3.2 Immunofluorescence

Frozen sections were thawed to room temperature, subjected to antigen retrieval, and permeabilized with 0.3–1% Triton X-100 for 30 min. After washing with PBS, sections were blocked with 10% goat serum for 30 min and incubated overnight at 4 °C with primary antibodies: Aβ (1:100, rabbit, Proteintech), NeuN (1:1000, rabbit, Abcam), and GFAP (1:400, rat, Thermo Fisher). The following day, sections were incubated at room temperature for 2 h with Alexa Fluor 594-conjugated secondary antibodies (1:400, Invitrogen). After nuclei staining with DAPI, slides were mounted with antifade medium. Images were captured using fluorescence microscopy.

### 2.4 Real-time quantitative reverse transcription PCR (RT-qPCR)

Hippocampal tissue was homogenized in RNA lysis buffer. Total RNA was extracted using TRIzol reagent (Thermo Fisher Scientific) and subsequently reverse transcribed with the RevertAid First Strand cDNA Synthesis Kit (Takara). Quantitative real-time PCR (RT-qPCR) was performed using 2 × RealStar Fast Probe qPCR Premix, gene-specific primers (Table 1), probes, and nuclease-free water. Thermal cycling conditions included an initial 2-min denaturation at 95 °C, followed by 35 cycles of 15 s at 95 °C for denaturation and 30 s at 60 °C for annealing/extension.

**Table 1:** mRNA primer sequences.

Gene name	Sequences
BCL2-F	GCTACCGTCGTGACTTCGC
BCL2-R	CCCCACCGAACTCAAAGAAGG
SPHK1-F	ACTGATACTCACCGAACGGAA
SPHK1-R	CCATCACCGGACATGACTGC
GAPDH-F	GCCAAGGCTGTGGGCAAGGT
GAPDH-R	TCTCCAGGCGGCACGTCAGA
iNOS-F	CACCAAGCTGAACTTGAGCG
iNOS-R	CGTGGCTTTGGGCTCCTC
IL-1β-F	TGGGAAACAACAGTGGTCAGG
IL-1β-R	CCATCAGAGGCAAGGAGGAA
IFN-γ-F	GAACTGGCAAAAGGATGGTGA
IFN-γ-R	TGTGGGTTGTTGACCTCAAAC
TNF-α-F	GACGTGGAACTGGCAGAAGAG
TNF-α-R	TTGGTGGTTTGTGAGTGTGAG
CXCL2-F	GCCCAGACAGAAGTCATAGCC
CXCL2-R	CTCCTCCTTTCCAGGTCAGTTA
Caspase1-F	CGTACACGTCTTGCCCTCAT
Caspase1-R	CTCTTTCACCATCTCCAGAGC

All primer sequences are listed in the 5′–3′ direction with GAPDH serving as the internal reference gene.

### 2.5 Malondialdehyde (MDA) assay

The concentration of MDA in hippocampal tissue homogenates was measured using a colorimetric assay kit (S0131S, Beyotime, China) according to the manufacturer’s instructions. Thiobarbituric acid (TBA) working solutions and serially diluted standards were prepared. After centrifugation, sample supernatants were collected, and absorbance was measured. MDA concentrations were calculated from a standard curve and expressed in molar units.

### 2.6 Statistical analysis

Image and data analyses were performed using GraphPad Prism 8.0.1 and ImageJ 1.8.0 software. Data are presented as mean ± SD. Normality was assessed using the Shapiro–Wilk test, and homogeneity of variances was evaluated with Levene’s test. Data with a normal distribution were analyzed by one-way ANOVA for multiple group comparisons and Student’s *t*-test for pairwise comparisons. Non-normally distributed data were analyzed using the Kruskal–Wallis *H* test or the Mann–Whitney *U* test. Welch’s *t*-test was used when variances were unequal. A two-tailed *p*-value of less than 0.05 was considered statistically significant. Sample sizes varied across assays due to pre-specified exclusions, as indicated in the figure legends. Behavioral testing and histological quantification were performed by investigators blinded to group allocation.

## 3 Results

### 3.1 Dexmedetomidine alleviates anxiety-like behaviors and cognitive deficits in AD mice

**Figure 1:**
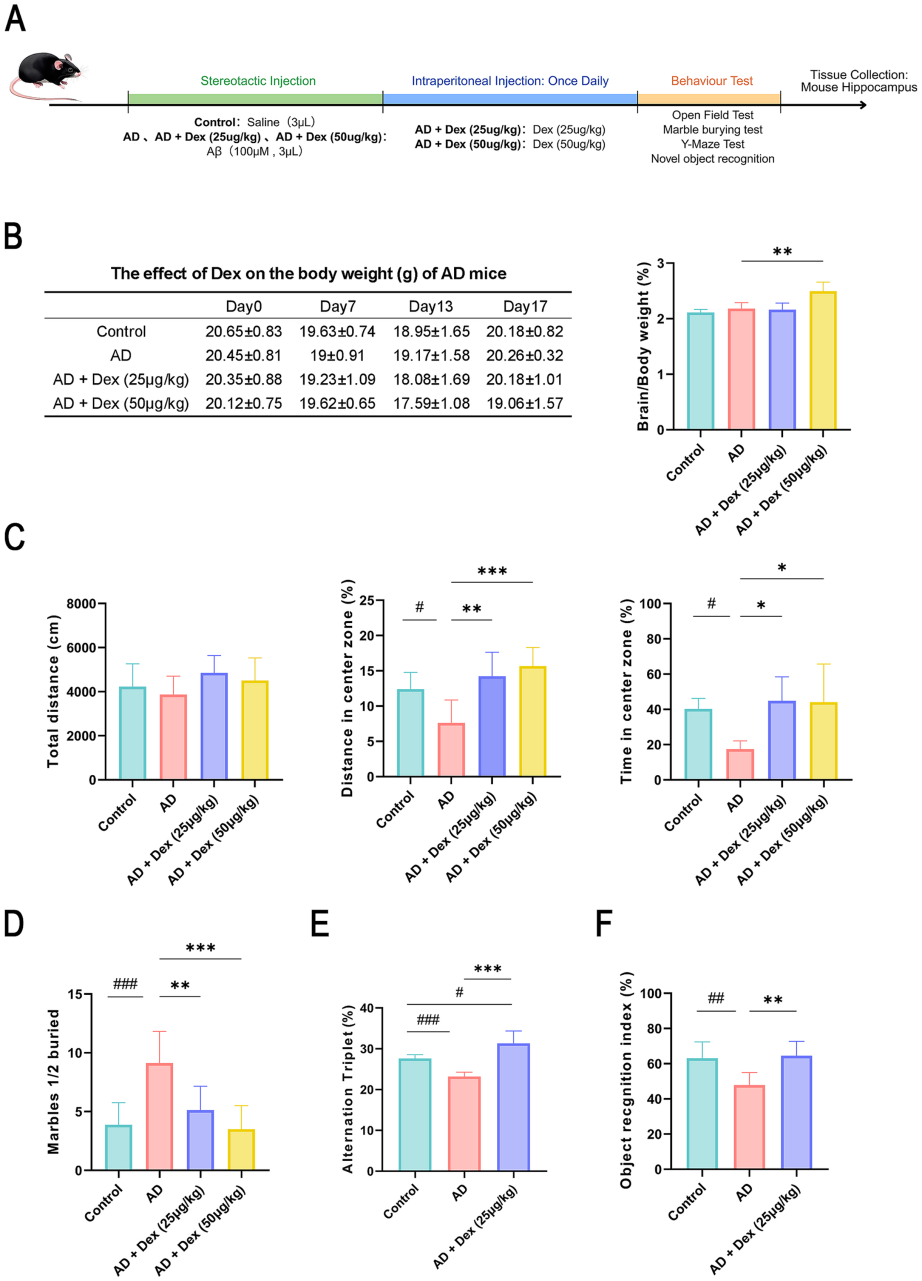
Dexmedetomidine alleviates anxiety-like behaviors and cognitive deficits in a mouse model of Alzheimer’s disease. **(A)** Experimental schedule. On day 0, oligomeric Aβ_1–42_ was delivered into the lateral ventricles via intracerebroventricular injection. Starting on day 7, mice were randomly divided into four groups: Control group, AD group, AD treated with Dex at 25 μg/kg, and AD treated with Dex at 50 μg/kg. Each group was administered daily intraperitoneal injections of either Dex or vehicle for seven consecutive days. **(B)** Body weight was tracked from day 0 to day 17 (Control: *n* = 8; Dex-treated groups: *n* = 13). Final bar graph shows brain-to-body weight ratios on day 17 (*n* = 5 for each group). **p < 0.01. **(C)** Behavioral metrics assessed in the open- field test included total travel distance, distance traveled in the center zone, and duration spent in the center area (*n* = 6 for each group). #*p* < 0.05, *p < 0.05, **p < 0.01, ***p < 0.001. **(D)** Total number of marbles buried per group in the marble burying test (*n* = 8 for each group). **p < 0.01, ###*p* < 0.001, ***p < 0.001. **(E)** Percentage of spontaneous alternations in the Y-maze, indicating working memory performance (*n* = 6 for each group). #*p* < 0.05, ###*p* < 0.001, ***p < 0.001. **(F)** Preference for novelty was quantified using the recognition index (RI) during the novel object recognition test (*n* = 8 for each group), **p < 0.01, ##*p* < 0.01. All experiments were performed in three independent replicates.

The overall experimental timeline is shown in Figure 1A. Body and brain weights were systematically recorded throughout the study (Figure 1B). Mice treated with Dex at doses of 25 or 50 μg/kg exhibited a more pronounced weight reduction compared to the Control and AD groups. After discontinuation of Dex administration, body weights gradually returned to baseline levels in all groups. By day 17, no significant differences in final body weight were observed between the Control, AD, and AD + Dex (25 μg/kg) groups, while the AD + Dex (50 μg/kg) group maintained a sustained reduction. Notably, brain weight was significantly higher in the AD + Dex (50 μg/kg) group than in the other groups.

Behavioral and histological assessments were performed to evaluate the anxiolytic and cognitive effects of Dex in AD mice. In the open-field test, no significant differences in total locomotor activity were observed across groups, suggesting that Aβ or Dex did not affect general mobility. However, AD mice spent less time and traveled shorter distances in the center zone compared to Control mice (*p* < 0.05), indicating heightened anxiety-like behavior. Dex treatment significantly ameliorated this phenotype, as reflected by increased exploration of the center zone (*p* < 0.05, Figure 1C).

Similarly, marble-burying behavior, a proxy for anxiety, was significantly increased in AD mice (*p* < 0.05), and substantially reduced following Dex treatment (*p* < 0.05, Figure 1D). The Y-maze test revealed a significant improvement in spontaneous alternation performance in the AD + Dex (25 μg/kg) group compared to the AD group (*p* < 0.001, Figure 1E), indicating enhanced working memory. Likewise, the novel object recognition test showed that Dex-treated mice spent more time exploring the novel object compared to AD mice (*p* < 0.05, Figure 1F), suggesting improvements in hippocampus-dependent recognition memory.

**Figure 2:**
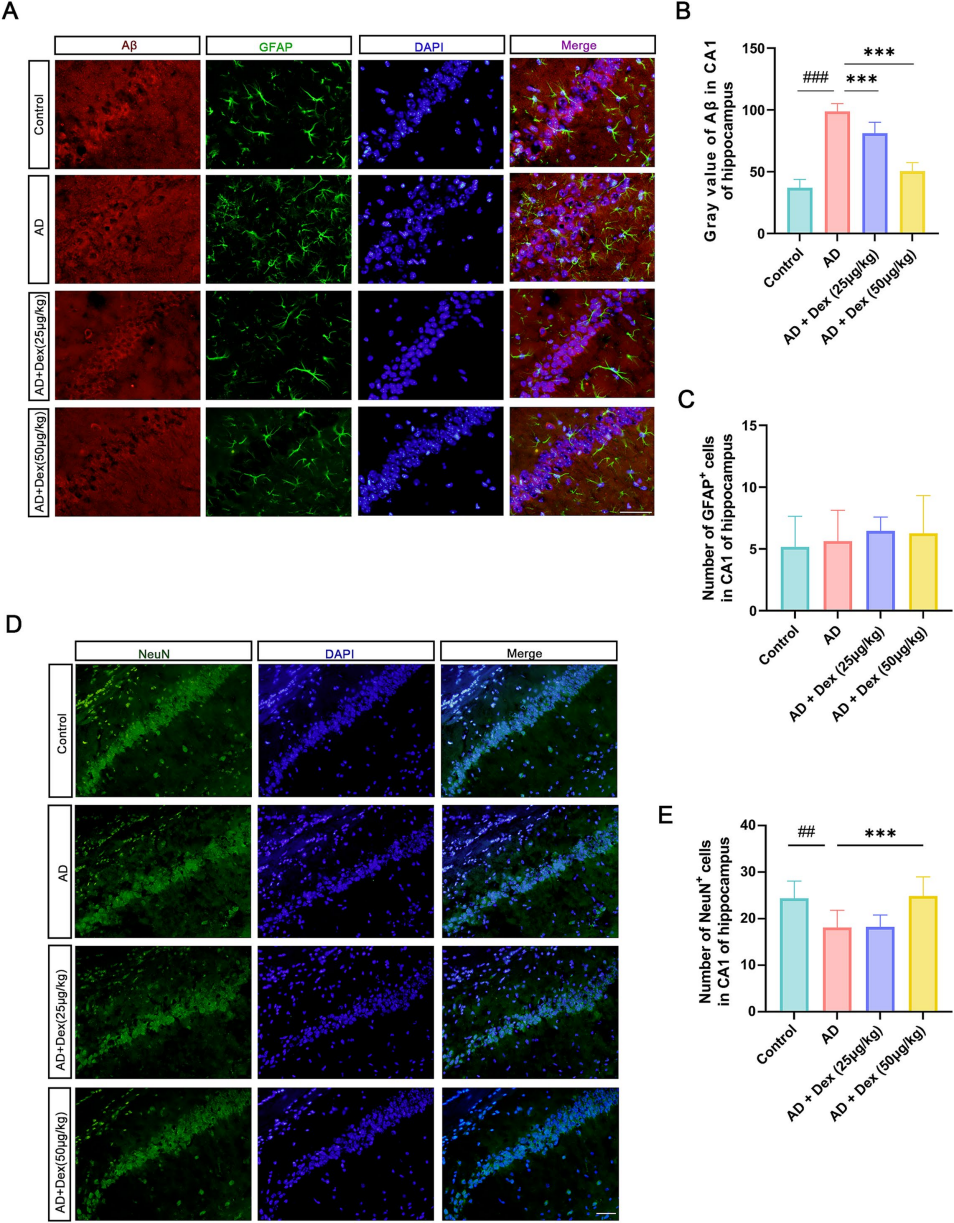
Dexmedetomidine attenuates hippocampal neuronal damage in AD mice. **(A)** Immunofluorescence staining showing Aβ_1–42_ plaques (red), GFAP-labeled astrocytes (green), DAPI (blue) marked nuclei in hippocampal CA1. **(B)** Fluorescence intensity of Aβ_1-42_ immunoreactivity quantified within the CA1 subregion of the hippocampus. ###*p* < 0.001, ***p < 0.001. **(C)** Number of GFAP-positive astrocytes per section in CA1 (*n* = 3 in each group). **(D)** NeuN immunostaining (green) and DAPI counterstaining (blue) visualizing hippocampal neurons. **(E)** Number of NeuN-positive neurons per CA1 section (*n* = 3 in each group), ##*p* < 0.01, ***p < 0.001. Scale bar = 50 μm. All experiments were performed in three independent replicates.

To explore mechanisms underlying these behavioral improvements, immunofluorescence staining was performed on the hippocampal CA1 region. AD mice exhibited significantly higher Aβ fluorescence intensity compared to Control mice (*p* < 0.001, Figure 2A), which was substantially reduced following Dex treatment at both low and high doses (*p* < 0.001, Figure 2B). No significant differences in the number of GFAP-positive astrocytes were observed among groups (Figure 2C). However, the number of neurons in the CA1 region was significantly reduced in AD mice compared to controls (*p* < 0.01, Figure 2D), and notably restored in the AD + Dex (50 μg/kg) group following treatment (*p* < 0.001, Figure 2E), indicating that Dex offers neuroprotection against Aβ-induced neuronal damage.

### 3.2 Dexmedetomidine attenuates neuroinflammation and normalizes aberrant neurogenesis in the dentate gyrus of AD mice

**Figure 3:**
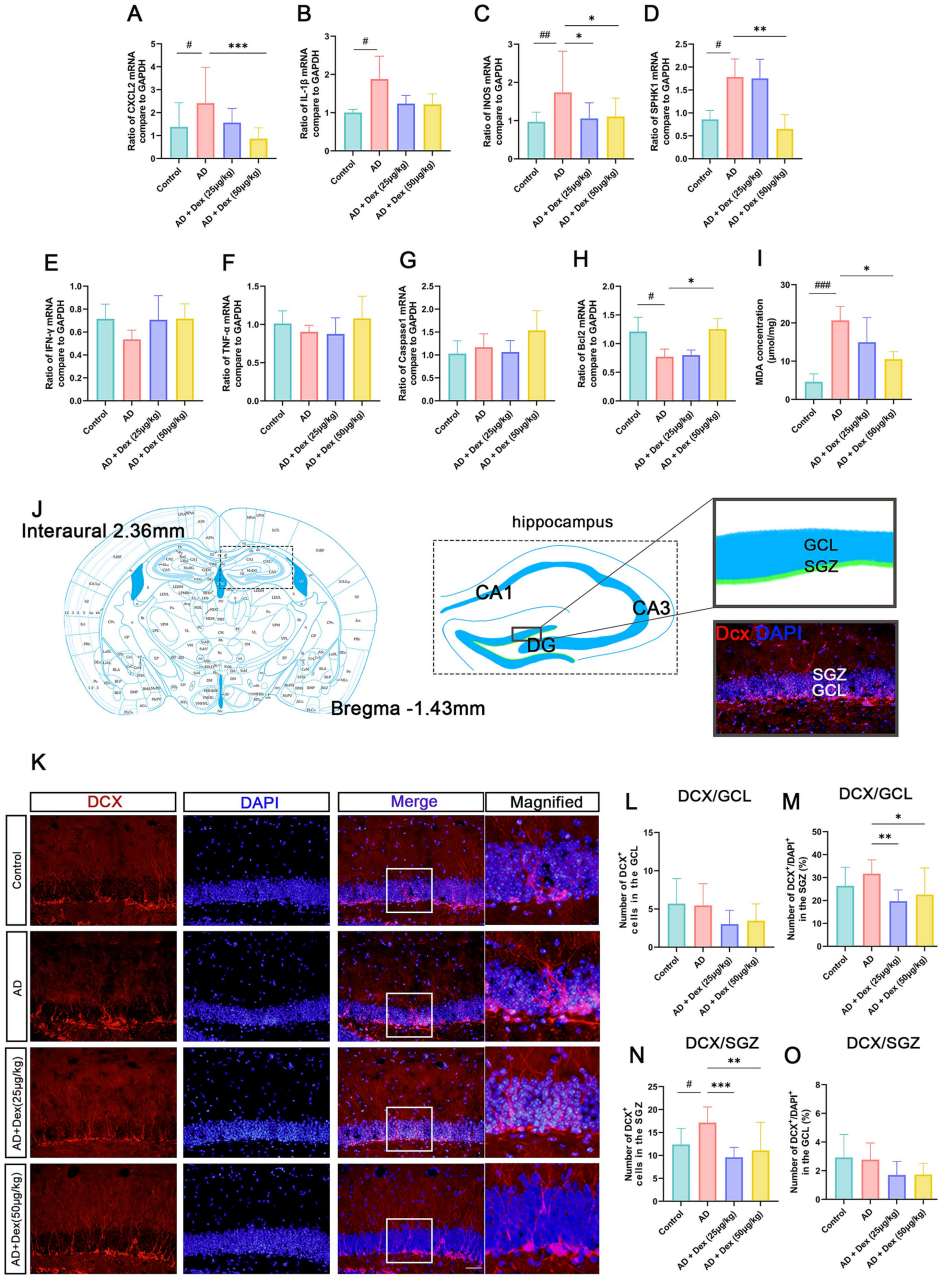
Dexmedetomidine alleviates neuroinflammation and normalizes aberrant neurogenesis in the dentate gyrus of AD mice. **(A–H)** mRNA expression levels of inflammatory and apoptotic markers (CXCL2, iNOS, IL-1β, SPHK1, IFN-*γ*, TNF-*α*, Caspase 1, and Bcl2) in hippocampal tissue, determined by qRT-PCR. GAPDH was used as the internal control. **(I)** ELISA-based quantification of malondialdehyde (MDA) levels in hippocampal lysates (*n* = 4 in each group). #*p* < 0.05, ##*p* < 0.01, ###*p* < 0.001, **p < 0.01, ***p < 0.001. **(J)** Schematic of the hippocampal region analyzed for DCX immunofluorescence. **(K)** Representative images of DCX-positive neurons (red) and DAPI-stained nuclei (blue) in the dentate gyrus. **(L–O)** Quantification of DCX-labeled cells in the subgranular zone (SGZ) and granule cell layer (GCL) per section (*n* = 3 in each group). #*p* < 0.05, *p < 0.05, **p < 0.01, ***p < 0.001. Scale bar = 50 μm. All experiments were performed in three independent replicates.

To assess hippocampal inflammation, mRNA expression levels of key inflammatory genes were measured. Compared with the control group, AD mice exhibited significantly elevated mRNA levels of CXCL2, iNOS, IL-1β, and SPHK1 (*p* < 0.05). Administration of Dex led to marked suppression of CXCL2, iNOS, and SPHK1 expression (*p* < 0.05). Notably, Bcl2 expression, which was reduced in the AD group (*p* < 0.05), was restored by 50 μg/kg Dex treatment (*p* < 0.05). No statistically significant alterations were detected in IFN-*γ*, TNF-*α*, or Caspase-1 expression among the experimental groups (Figures 3A–H).

To evaluate lipid peroxidation and oxidative stress, MDA levels were measured. AD mice exhibited elevated MDA concentrations compared to controls (*p* < 0.05), which were significantly reduced by Dex treatment at both 25 μg/kg and 50 μg/kg doses (*p* < 0.05, Figure 3I). These results suggest that inflammation-driven oxidative damage contributes to neuronal injury in the AD model.

In adult mice, neurogenesis primarily occurs in the SGZ of the dentate gyrus. To determine whether Dex influences this process, the number of doublecortin (DCX)-positive immature neurons in the dentate gyrus was quantified (Figures 3J,K). AD mice exhibited a significant increase in DCX-labeled cells compared to controls (*p* < 0.05), indicating aberrant neurogenesis. Dex treatment significantly reduced the number of these newborn neurons in both the 25 μg/kg and 50 μg/kg groups (*p* < 0.05, Figures 3L–O), consistent with normalization of aberrant neurogenesis.

These results strongly support the hypothesis that AD-related neuroinflammation promotes abnormal neurogenesis in the adult dentate gyrus. Additionally, Dex effectively mitigates this process, likely contributing to its neuroprotective effects.

### 3.3 Yohimbine reverses the cognitive and neuroprotective effects of dexmedetomidine in AD mice

**Figure 4:**
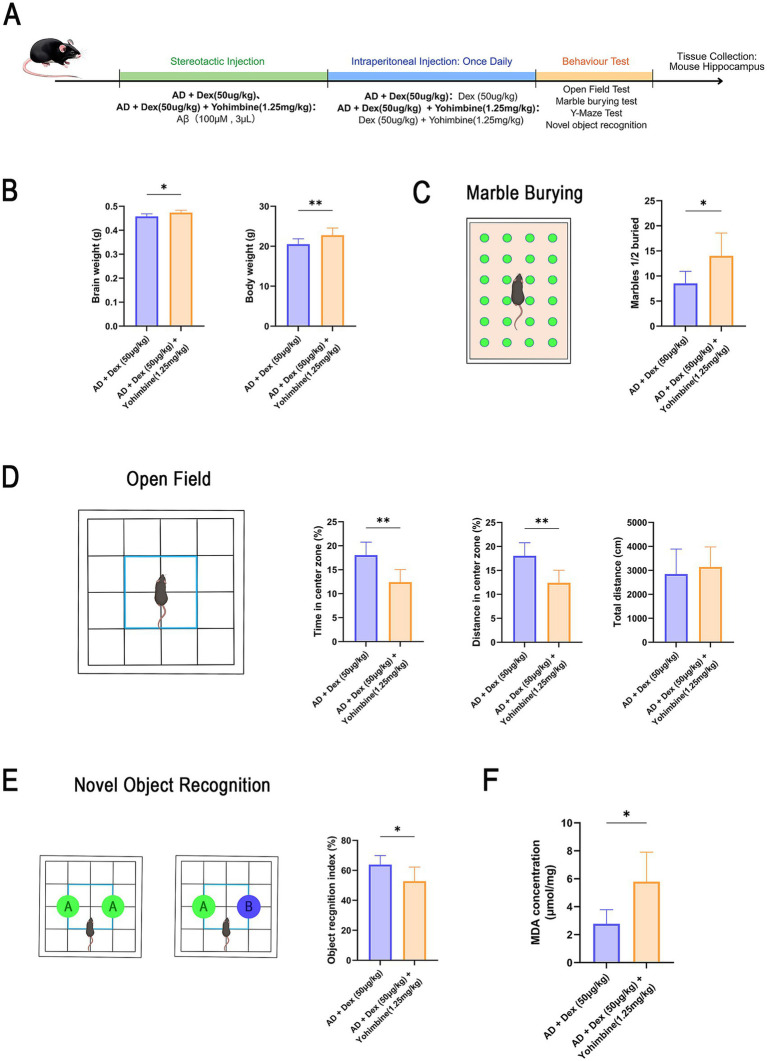
Yohimbine reverses the cognitive and behavioral benefits of Dexmedetomidine in AD mice. **(A)** Experimental schematic. Mice received intracerebral Aβ injection on day 0. On day 7, animals were allocated to AD + Dex (50 μg/kg) or AD + Dex (50 μg/kg) + Yohimbine (1.25 mg/kg) groups. Dex (50 μg/kg) was administered daily, and Yohimbine (1.25 mg/kg) was injected 30 min post-Dex for 7 days. **(B)** Body weight on day 17 and corresponding brain-to-body weight ratios (*n* = 5 in each group), *p < 0.05, **p < 0.01. **(C)** Model diagrams of Marble-Burying Test. Number of marbles buried in each group as a measure of anxiety-related behavior [AD + Dex (50 μg/kg): *n* = 6; AD + Dex (50 μg/kg) + Yohimbine (1.25 mg/kg): *n* = 8], *p < 0.05. **(D)** Model diagrams of open-field test. Open-field test data showing total distance, mean velocity, center distance, and time in the center zone [AD + Dex (50 μg/kg): *n* = 10; AD + Dex (50 μg/kg) + Yohimbine (1.25 mg/kg): *n* = 9], **p < 0.01. **(E)** Schematic of novel object recognition test. Recognition index (RI) in novel object recognition test [AD + Dex(50 μg/kg): *n* = 6; AD + Dex(50 μg/kg) + Yohimbine (1.25 mg/kg): *n* = 8], *p < 0.05. **(F)** MDA concentrations in hippocampal tissue measured via ELISA [AD + Dex(50 μg/kg): *n* = 6; AD + Dex(50 μg/kg) + Yohimbine (1.25 mg/kg): *n* = 4], *p < 0.05. All experiments were performed in three independent replicates.

The experimental design for the Yohimbine cohort is summarized in Figure 4A. To assess whether α_2_-adrenergic signaling mediates the therapeutic effects of Dex, Yohimbine, a selective α_2_-adrenergic receptor antagonist, was co-administered to AD mice. Yohimbine co-treatment significantly increased both body and brain weights compared to Dex treatment alone (*p* < 0.05, Figure 4B), suggesting a reversal of Dex-induced physiological changes.

Behavioral assessments showed that Yohimbine abolished the anxiolytic and cognitive benefits of Dex. In the open-field test, total locomotor activity remained unchanged between groups, indicating no impact on gross motor function. However, center zone activity was reduced following Yohimbine treatment (*p* < 0.05, Figure 4D), indicating a return of anxiety-like behavior. Similarly, marble-burying behavior was significantly elevated (*p* < 0.05, Figure 4C), and the time spent exploring the novel object was significantly shorter. (*p* < 0.05, Figure 4E), reflecting a reversion to cognitive impairment.

Biochemical analysis demonstrated that Dex-mediated reductions in oxidative stress were reversed by Yohimbine, as indicated by elevated MDA levels in the AD + Dex(50 μg/kg) + Yohimbine (1.25 mg/kg) group (*p* < 0.05, Figure 4F).

**Figure 5:**
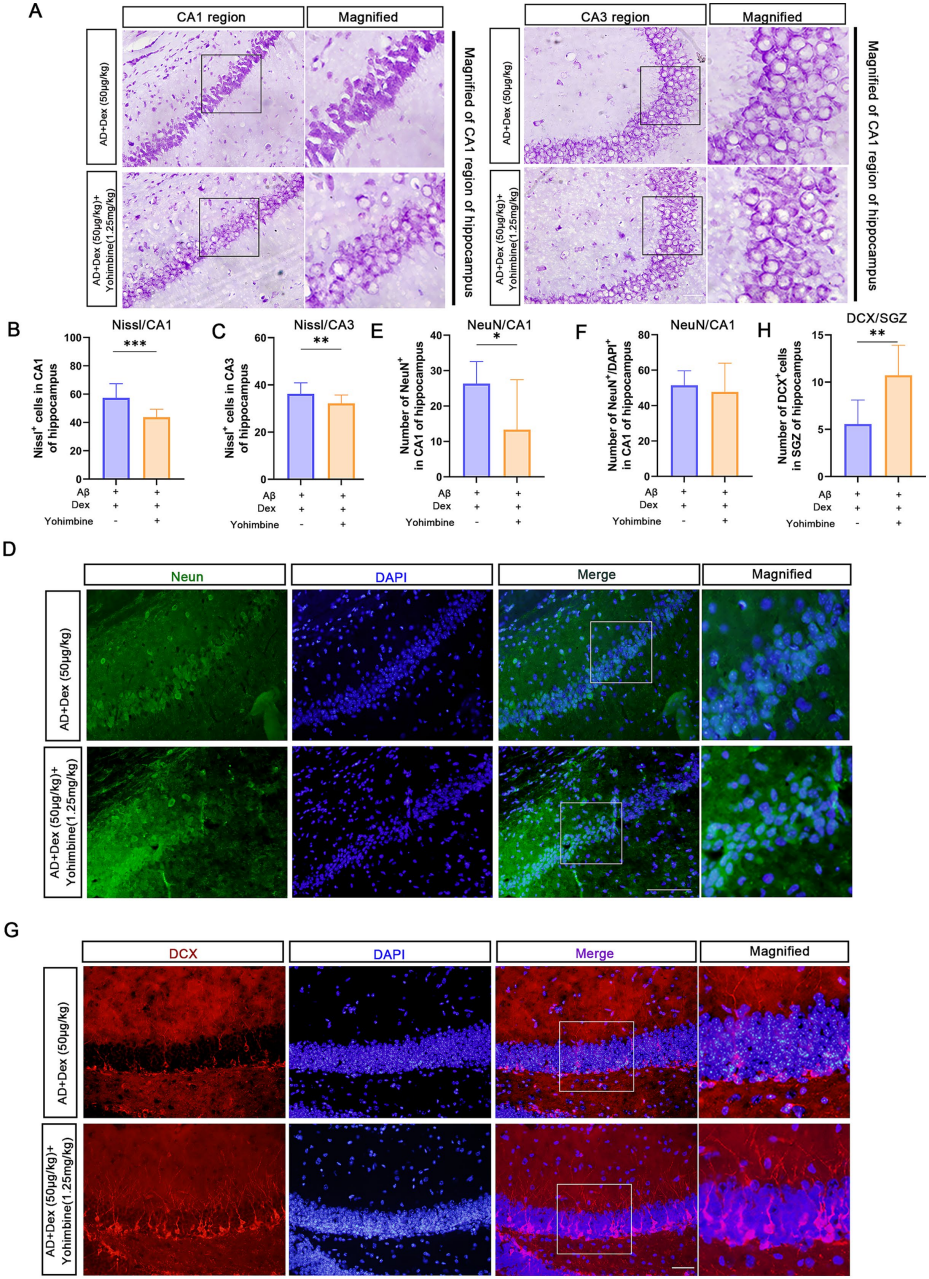
Yohimbine abolishes the neuroprotective effects of Dexmedetomidine in AD mice. **(A)** Nissl staining showing neuronal populations in CA1 and CA3 regions of the hippocampus. **(B,C)** Quantification of Nissl-positive cells in CA1 and CA3 regions per group, (*n* = 3 mice in each group), **p < 0.01, ***p < 0.001. **(D)** NeuN (green) and DAPI (blue) staining of hippocampal neurons. **(E,F)** Quantification of NeuN-positive neuronal cells per section in the hippocampal CA1 region. (*n* = 3 mice in each group), *p < 0.05. **(G)** Immunofluorescent labeling of doublecortin (DCX, red) and DAPI (blue) to visualize newly generated neurons within the dentate gyrus. **(H)** Quantitative analysis of DCX-labeled cells within the subgranular zone (SGZ) per coronal brain section. (*n* = 3 mice in each group), **p < 0.01. Scale bar = 50 μm. All experiments were performed in three independent replicates.

Histological analysis revealed that Yohimbine attenuated the neuroprotective effects of Dex in the CA1 region of the hippocampus. Animals co-treated with Dex and Yohimbine exhibited a notable reduction in Nissl-positive cell numbers (*p* < 0.05, Figures 5A–C) and had significantly fewer CA1 neurons than those receiving Dex alone (*p* < 0.05, Figures 5D–F).

DCX immunostaining further revealed increased neurogenesis in the SGZ following Yohimbine treatment, evidenced by a significant rise in newborn neuron numbers compared to the AD + Dex (50 μg/kg) group (*p* < 0.01, Figures 5G,H), suggesting a reactivation of aberrant neurogenesis.

Collectively, these results emphasize the α2-adrenergic receptor as a key mediator of Dex’s neuroprotective, anti-inflammatory, and cognitive-enhancing effects in AD pathology, and that these effects can be effectively antagonized by Yohimbine.

## 4 Discussion

Alzheimer’s disease is characterized by progressive neurodegeneration, with hallmark pathological features including extracellular Aβ plaques and intracellular tau neurofibrillary tangles (Knopman et al., 2021). Clinically, AD presents with cognitive decline, mood instability, and behavioral dysregulation. The accumulation of Aβ peptides triggers a cascade of neurotoxic events, compromising neuronal integrity and activating microglia, which in turn secrete pro-inflammatory mediators that drive chronic neuroinflammation and exacerbate neuronal damage (Ma et al., 2022; Uddin et al., 2022).

In the hippocampus, the SGZ of the dentate gyrus sustains lifelong neurogenesis through self-renewing NSCs, which are essential for learning, memory, and emotional regulation (Drew et al., 2013; Gonçalves et al., 2016; Seong et al., 2016; Niklison-Chirou et al., 2020). Disruption of this neurogenic process has been mechanistically linked to neuroinflammatory signaling, which promotes aberrant NSC proliferation and differentiation (Sung et al., 2020; Tan et al., 2022; Zeng et al., 2023). Consequently, anti-inflammatory and antioxidant therapies have emerged as promising strategies to restore neurogenic balance and mitigate cognitive deficits in AD.

Dex, a selective α_2_-adrenergic receptor agonist, has demonstrated both neuroprotective and anti-inflammatory properties in various experimental models (Gao et al., 2019; Qiu et al., 2020). Dex has been reported to mitigate oxidative stress, including lower MDA levels, and to reduce postoperative delirium across multiple preclinical models (Pandharipande et al., 2007; Schoeler et al., 2012; Wang et al., 2014; Su et al., 2016). However, its precise role in modulating AD-related neuropathology has not been fully explored.

The findings of this study indicate that Dex treatment enhances cognitive performance and reduces anxiety-like phenotypes in an AD mouse model. Treated animals showed improved spontaneous alternation in the Y-maze and increased exploration of novel objects, consistent with previous findings (Zhang et al., 2023). Additionally, Dex treatment alleviated anxiety-related behaviors, evidenced by enhanced center zone activity in the open-field test and reduced marble-burying behavior. The comparable improvements produced by both the 25 and 50 μg/kg doses of Dex in most behavioral assays, with the exception of the Y-maze, likely reflect the nonlinear dose–response characteristics of glucocorticoid-related signaling. Both doses may fall within an effective range that enhances hippocampal-dependent working memory, leading to similar behavioral outcomes. The similar efficacy observed at the higher dose across additional behavioral paradigms further indicates that distinct cognitive and pathological domains may possess their own optimal therapeutic thresholds. Supporting this interpretation, previous studies have demonstrated an inverted U-shaped glucocorticoid dose–response relationship, in which low-to-moderate levels enhance memory consolidation, whereas higher levels provide diminished benefits or even impair performance (Roozendaal and McGaugh, 1996; Roozendaal et al., 1999; de Quervain et al., 2009). Systematic evaluation of additional Dex doses will be necessary to more precisely define the nonlinear profile underlying its cognitive and neuroprotective effects.

Histological analysis showed that Dex attenuated neuronal loss in the hippocampal CA1 region, a principal output node with strong connectivity to limbic circuits that regulate emotion and anxiety. By contrast, SGZ of the dentate gyrus serves as the primary neurogenic niche of the adult hippocampus and supports input-stage plasticity through ongoing neurogenesis. Together, CA1 and the SGZ form functionally interconnected components of the trisynaptic hippocampal pathway. We therefore examined both regions to capture complementary dimensions of hippocampal repair: CA1 neuronal survival reflects the integrity of the output pathway, whereas SGZ neurogeness indexes the regenerative potential of the input stage. This region-specific approach provides a coherent framework for interpreting the multifaceted effects of Dex on hippocampal structure and function (Hainmueller and Bartos, 2020; Fölsz et al., 2023).

Dex appears to exert its therapeutic effects in AD through anti-inflammatory and neuroprotective mechanisms that display clear dose-dependent properties. Prior studies indicate that lower Dex doses provide modest metabolic or anti-inflammatory modulation, whereas higher doses more reliably activate neuroprotective cascades and preserve vulnerable CA1 pyramidal neurons (Dahmani et al., 2008; Hou et al., 2020). In this context, the 25 μg/kg dose may fall below the threshold required for structural rescue in CA1, while 50 μg/kg achieves sufficient receptor engagement to produce measurable neuroprotection. This dose–response pattern is consistent with findings from other Dex-treated neural injury models. Notably, both doses improved behavioral outcomes, suggesting that cognitive recovery depends on broader neural processes that are less tightly dose-sensitive than CA1 neuronal preservation. At the molecular level, Dex-mediated reductions in CXCL2, iNOS, and SPHK1 further support its ability to restrain microglial activation and cytokine-driven neurotoxicity in AD, offering a mechanistic explanation for the dose-dependent neuroprotection observed in CA1.

Interestingly, similar anti-inflammatory effects have been observed with donepezil, an established AD treatment, through the suppression of COX-2, IL-1β, IL-6, and iNOS (Kim et al., 2021). Our findings extend this paradigm by showing that Dex reduces Aβ deposition in the hippocampus, aligning with clinical observations that reduced Aβ burden leads to improved cognitive and functional outcomes (van Dyck et al., 2023). Mechanistically, Dex may influence LAMP5-related hyperexcitability and enhance neuronal activation (Deng et al., 2022); however, the role of tau was not assessed in this study, and potential tau-related effects remain to be explored. Furthermore, elevated cytokine levels have been shown to facilitate Aβ release and impair its glial degradation, accelerating disease progression (Seong et al., 2016).

Thus, Dex appears to exert therapeutic effects in AD through a multifaceted mechanism that includes decreasing Aβ burden, preserving neuronal integrity, and modulating inflammation. These actions contribute to the restoration of cognitive function and emotional regulation. The pharmacological blockade with Yohimbine, an *α*_2_-adrenergic receptor antagonist, reversed Dex’s effects, highlighting the critical role of α_2_-receptor signaling in mediating its therapeutic actions.

Although our pharmacological findings with the α₂-adrenergic antagonist Yohimbine support a role for α₂-adrenergic receptors in mediating the effects of Dex, we did not directly examine the downstream signaling pathways responsible for these actions. Accumulating evidence indicates that canonical intracellular cascades, including PI3K/Akt, MAPK/ERK and NF-κB, contribute to Dex-induced protection across diverse experimental models. For example, Dex has been reported to attenuate neuroinflammation and cognitive impairment by suppressing HMGB1/RAGE/NF-κB signaling (Wang et al., 2022) and to reduce apoptosis and improve tissue survival after ischemia/reperfusion injury via activation of PI3K/Akt and STAT3 (Chang et al., 2020; Chen et al., 2023). These observations raise the possibility that similar mechanisms underlie the neuroprotective effects observed in our Aβ₁–₄₂ model. Future work systematically assessing PI3K/Akt, MAPK/ERK and NF-κB pathway activation after Dex treatment will be essential to delineate the precise molecular mediators of its actions.

In addition to these mechanistic considerations, the translational relevance of our findings warrants further discussion. The present study was performed in male mice, which enabled a controlled evaluation of Aβ₁–₄₂–induced pathology but does not fully capture the biological heterogeneity observed in clinical Alzheimer’s disease. Aging is known to amplify neuroinflammatory reactivity and accelerate Aβ-driven neuropathology, while sex-related differences in immune signaling and vulnerability to neurodegeneration have been increasingly recognized. These factors may influence both disease progression and responsiveness to Dex (Schmid et al., 2017; Hendrickx et al., 2022; Luo et al., 2024; Rosenzweig et al., 2024). Therefore, future studies incorporating aged animals and female cohorts will be essential to determine whether the therapeutic effects observed here extend to populations that more closely model human AD.

In conclusion, these findings position Dex as a promising modulator of neuroinflammatory and amyloidogenic processes in AD. Its ability to simultaneously address pathological, behavioral, and molecular aspects of the disease underscores its translational potential for managing both cognitive decline and neuropsychiatric symptoms in AD.

## 5 Limitations and perspectives

This study provides evidence supporting the neuroprotective role of Dex in an AD mouse model, particularly through its modulation of neuroinflammation and aberrant hippocampal neurogenesis. However, several limitations should be acknowledged. First, although the acute Aβ₁–₄₂ model is widely used to investigate mechanisms of amyloid-induced neurotoxicity, it does not reproduce key features of AD such as tau pathology and chronic neuroinflammation (Yokoyama et al., 2022). Thus, our findings primarily reflect early, amyloid-driven changes rather than the full spectrum of AD pathology. Future studies using chronic models or models that combine amyloid and tau pathology will be essential to determine whether these mechanisms generalize to later and more complex stages of the disease. Second, all experiments in this study were performed in male mice. This choice minimizes variability related to the estrous cycle and improves consistency across experiments, but it also restricts the generalizability of our findings. Previous work has shown that sex influences hippocampal signaling, neural progenitor dynamics, microglial activation, and inflammatory responses (Spencer-Segal et al., 2011; Hanamsagar and Bilbo, 2016; Zonis et al., 2018; Han et al., 2021; Britton et al., 2022), raising the possibility that females may respond differently in AD models. Future studies that include female animals will be necessary to determine whether the neuroinflammatory and neurogenic effects of Dex observed here are sex-dependent. Third, the short treatment duration limits our understanding of Dex’s long-term efficacy and potential adverse effects, especially in aged animals where neuroplasticity and drug sensitivity may differ. Future investigations using transgenic AD models that better mimic human disease progression, combined with extended treatment paradigms, are needed to clarify the therapeutic window and safety profile of Dex. Additionally, the effects of Dex on glial phenotypes, the neurogenic niche, and synaptic remodeling remain incompletely understood and warrant further exploration. These findings may ultimately help refine Dex-based strategies for early AD intervention and support its rational repurposing in neurodegenerative disorders.

## Data Availability

The raw data supporting the conclusions of this article will be made available by the authors, without undue reservation.
